# Exploiting residual cocoa biomass to extract advanced materials as building blocks for manufacturing nanoparticles aimed at alleviating formation-induced oxidative stress on human dermal fibroblasts†

**DOI:** 10.1039/d4na00248b

**Published:** 2024-05-30

**Authors:** Joel Girón-Hernández, Yeison Barrios Rodríguez, Noemi Corbezzolo, Dayana Orozco Blanco, Carlos Carranza Gutiérrez, William Cheung, Piergiorgio Gentile

**Affiliations:** a Department of Applied Sciences, Faculty of Health and Life Sciences, Northumbria University NE1 8ST Newcastle Upon Tyne UK joel.l.g.hernandez@northumbria.ac.uk; b i-Food, Instituto Universitario de Ingeniería de Alimentos-FoodUPV, Universitat Politècnica de València 46021 Valencia Spain; c Centro Surcolombiano de Investigación en Café (CESURCAFÉ), Universidad Surcolombiana 410010 Neiva Colombia; d School of Engineering, Newcastle University NE1 7RU Newcastle Upon Tyne UK Piergiorgio.gentile@ncl.ac.uk; e Escuela de Ciencias Agrícolas, Pecuarias y del Medio Ambiente, Universidad Nacional Abierta a Distancia 111511 Bogotá Colombia

## Abstract

The global adoption of by-product valorisation processes aligns with the circular economy framework, ensuring sustainability in the agricultural sector. In cocoa production, residual biomass can offer the opportunity to extract advanced materials, contributing to nanotherapeutic solutions for biomedical applications. This study explores extraction processes for valorising cocoa pod husks (CPHs) and optimising valuable cocoa-derived biocompounds for enhanced health benefits. Various extraction processes are compared, revealing the significant influence of CPH powder amount and extraction time. Furthermore, metabolic analysis identifies 124 compounds in the metabolite mix, including tartaric acid, gluconic acid and bioactive agents with antioxidant properties, resulting in a high total phenolic content of 3.88 ± 0.06 mg g^−1^. Moreover, the extracted pectin, obtained through alkaline and enzymatic routes, shows comparable yields but exhibits superior antioxidant capacity compared to commercial pectin. The study progresses to using these extracted biocompounds to develop Layer-by-Layer multifunctionalised nanoparticles (LbL-MNPs). Physico-chemical characterisation *via ζ*-potential, FTIR-ATR, and XPS confirms the successful multilayer coating on mesoporous silica nanoparticles (MNPs). TEM analysis demonstrates a uniform and spherical nanoparticle morphology, with a size increase after coating. *In vitro* biological characterisation with neo-dermal human fibroblast cells reveals enhanced metabolic activity and biocompatibility of LbL-MNPs compared to bare MNPs. Also, the engineered nanoparticles demonstrate a protective effect against H_2_O_2_-induced intracellular oxidative stress on human dermal fibroblast cell lines, showcasing their potential as antioxidant carriers for biomedical applications.

## Abstract 2

Revalorising cocoa pod-husk biomass to extract biomolecules as building blocks for developing Layer-by-Layer functionalised-nanoparticles to alleviate formation-induced oxidative stress on human dermal fibroblasts.

## Graphical abstract

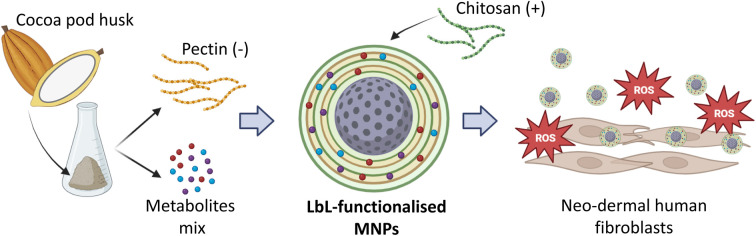

## 1. Introduction

Cocoa bean production plays a crucial role in the economies of many developing countries, but on a global scale, it largely follows a linear economic model, which threatens sustainability.^1^ The consumption of cocoa butter and liquor has driven an expansion in planting areas, consequently increasing the generation of residual biomass, which accounts for approximately 60–70% of fresh fruit.^2^ According to the International Cocoa Organisation, in 2022, a total of 16 072.2 tons of cocoa were traded globally, resulting in approximately 12 857.76 tons of residual biomass derived from post-harvest activities, including pod husks (CPHs), leachate and pulp.

Currently, cocoa plantations in producing countries lack management plans for processing the residual biomass generated during the post-harvest step, which is generally unexploited.^3^ Also, there are limited strategies dedicated to utilising this biomass for extracting biomolecules and studying their properties, with a notable deficiency of exploration regarding potential applications across various industries, including food, nutrition, cosmetics, and biomedicine. For example, the development of nano-advanced materials for therapeutic applications could foster innovation in the field of biomedical engineering by exploiting the extracted biomaterials from CPHs to address health-related issues in society.

Furthermore, extracting advanced materials and bioactive components from CPHs aligns with sustainability efforts in the cocoa industry, helping to reduce waste and maximise the use of cocoa by-products, promoting eco-friendly practices.^4^ Additionally, the literature reports several studies on cocoa bean processing while few investigations have been done on the pods' revalorisation. However, CPHs contain a significant number of antioxidant and anti-inflammatory compounds, including flavonoids and phenolic acids, which are gaining great interest due to their potential health benefits.^5^ For instance, the antioxidants in CPHs have the capability to neutralise harmful free radicals in the body and help reduce oxidative stress.^6^ Furthermore, from this biomass, biopolymers can be extracted including pectin (PEC), whose primary recognition comes from its role as a gelling agent in the food industry, particularly as a thickening agent.^2^ Also, PEC possesses anionic chemical groups capable of dissociating in aqueous solutions, making it a negatively charged polyelectrolyte. This characteristic allows it to interact with other charged molecules, enabling its use in the controlled release of bioactive components. Specifically, its polyelectrolyte properties have been harnessed for creating large microbeads in combination with chitosan (CH) reaching an average diameter of 120 μm. These microbeads were synthesised by injecting PEC solutions into a cross-linking bath followed by immersion in a CH bath.^7^ CH is a non-toxic polycationic derivative of chitin, biodegradable and biocompatible.^8^ Its high positive charge density makes it intrinsically antimicrobial, as the polyelectrolyte binds to and disrupts bacterial membranes. Recently, the electrostatic interaction between PEC and CH has been proposed to also form nanoscale coatings using the Layer-by-Layer (LbL) assembly technique.^9^ LbL, a cost-effective method with safe and simple features, effectively modifies surface properties, including flat films or particles. Notably, Martins *et al.*^10^ developed a PEC/CH multilayered coating on oxidised glass samples with durable and cytocompatible surfaces, exhibiting potent antiadhesive and antimicrobial activities against both *P. aeruginosa* and *S. aureus*.

In this scenario, this work investigates (1) the valorisation of CPH biomass to extract biomolecules as building blocks, and (2) the use of LbL nanotechnology to develop advanced functionalised nanoparticles for releasing a mixture of metabolites in a controlled and timely manner, thereby inducing specific biological activities. Thus, this work aligns with the circular economy principle by repurposing cocoa by-products into advanced materials with therapeutic potential. Specifically, due to the intrinsic antioxidant properties of extracted PEC and phenolics, we hypothesise that these functionalised advanced nanomaterials could alleviate formation-induced oxidative stress in cells. Particularly, reactive oxygen species (ROS), including hydrogen peroxide (H_2_O_2_), superoxide anions (O_2_^−^), and hydroxyl radicals (OH˙), are pivotal signalling molecules in many physiological processes and are often overproduced in various inflammatory tissues. ROS overproduction can disrupt cellular homeostasis, cause non-specific damage to critical components, and lead to various diseases.^11^ Therefore, an ideal biomaterial design strategy for regenerative medicine should aim to mitigate the effects of ROS produced in the body, providing a stable microenvironment for the differentiation of mesenchymal stem cells capable of producing new tissue.^12^ Thus, in this study, we optimise the extraction of valuable biocompounds, such as pectin and an extract containing a mix of metabolites. We employ design of experiments, utilising ethanol–water ultrasound assistance for extracting the metabolite mix, and alkaline and enzymatic solutions for recovering the pectin. Subsequently, these extracted biomaterials serve as components for manufacturing LbL functionalised mesoporous silica nanoparticles (LbL-MNPs). We then evaluate these nanosystems for their cytocompatibility and ability to suppress intracellular ROS induced by hydrogen peroxide in human dermal fibroblast cells, as a model of wound healing.

## 2. Experimental

### 2.1 Chemicals

CPH powder was provided by Universidad Surcolombiana (Neiva, Colombia). Caffeine 99%, theobromine 99%, Carrez I and II solutions, and trifluoroacetic acid 99% were purchased from Fisher Scientific, UK, while 1-phenyl-3-methyl-5-pyrazolone (PMP), sodium carbonate (pharma grade), l-ascorbic acid, and resveratrol were from Apollo Scientific, UK. Glacial acetic acid (ACS reagent, ≥99.7%), hydrochloric acid (ACS reagent, 37%), sodium chloride (ACS reagent, ≥99%), ethanol 96%, Dulbecco's Phosphate Buffer Saline (PBS), mesoporous silica nanoparticles (200 nm particle size and 4 nm pore size), sodium hydroxide (reagent grade, ≥98%), citrus pectin, chitosan (low molecular weight, deacetylation degree 75%), Folin & Ciocalteu′s phenol reagent, gallic acid monohydrate (ACS reagent, ≥98%), 2,2-diphenyl-1-picrylhydrazyl (DPPH), d-galacturonic acid (GalA), glucuronic acid (GlcA), galactose (Gal), glucose (Glc), mannose (Man), xylose (XFyl), rhamnose (Rha), arabinose (Ara), acetonitrile (ACN), analytical-grade methanol, ammonium bicarbonate, ammonium hydroxide and formic acid were supplied by Merck, UK. Suppliers for all the other materials were: cellulase complex Cellic® CTec2 (Novozymes, Denmark), nylon membrane Titan3 (0.45 μm) filters and Invitrogen™ Ferric Reducing Antioxidant Power (FRAP) assay kit (Thermo Scientific, UK), glass vials (Chromatography Direct, UK), 96 well plate Costar (Corning, USA), and half area 96 well microplate (Greiner Bio, Germany), and baking paper was purchased from Sainsbury, UK. Distilled water (dH_2_O) was obtained from a PureA-Q+ System (SLS-LabPro, UK).

### 2.2 Extraction of valuable components from CPHs

#### 2.2.1. Cocoa waste processing and preparation

Ripe and healthy cocoa fruits (*Theobroma cacao* L.) were randomly collected from a commercial plantation situated in a rural area of Rivera-Huila, Colombia. After harvesting, the fruits were washed and depulped to obtain the pod husks, which were then cut and subjected to a solar drying procedure. Subsequently, the prepared biomass was placed in an oven set at 40 °C for 24 h, ensuring moisture elimination before grinding. A MultiDrive basic mill (IKA, Germany) was employed to achieve a particle size of ∼200 microns. The resulting CPH powder was stored in plastic bags for subsequent valorisation.

#### 2.2.2. Design of experiments

To optimise the building block extraction recovered from the CPH powder, the response surface methodology was employed, utilising different designs based on the characteristics of the factors applied in each extraction. The metabolite mix (*via* an ethanol–water solution) and pectin (*via* an alkaline solution) were investigated with a central composite design, using the total phenolic content (mg g^−1^) of the extracts and pectin extraction yield (%) as response variables, respectively. The pectin extraction *via* the enzymatic route was investigated using a Box–Behnken design to avoid operating outside the recommended enzyme concentration range (1–10%) specified by the manufacturer. In this experiment, the selected response variable was the pectin extraction yield (%). All experimental setups for the extractions were run using Minitab software, version 21.4. Table 1 presents the ranges of the factors analysed in the extraction of the building blocks from the CPH powder.

**Table 1:** Range of experimental variables for the response surface designs applied to extract the building blocks from CPH powder

Component and route	Factor		Level				
			−*α*	−1	0	1	*α*
**Central composite design**							
Metabolite mix EtOH: H_2_O	*X*_1_	Solvent/CPH ratio (ml g^−1^)	9.9	15	20	30	35.1
	*X*_2_	Temperature (°C)	23.2	30	40	50	56.8
	*X*_3_	Extraction time (min)	16.5	25	37.5	50	58.5
Pectin alkaline	*X*_1_	Solvent/CPH ratio (ml g^−1^)	23.2	30	40	50	56.8
	*X*_2_	Temperature (°C)	43.2	50	60	70	76.8
	*X*_3_	Extraction time (min)	20.7	45	67.5	90	105.3

**Box–Behnken design**							
Pectin enzymatic	*X*_1_	Solvent/CPH ratio (ml g^−1^)	—	30	40	50	—
	*X*_2_	Enzyme/CPH ratio (%)	—	1	5.5	10	—
	*X*_3_	Extraction time (h)	—	6	15	24	—

#### 2.2.3. Extraction of the metabolite mix from CPH powder

Calculated amounts of CPH powder (*X*_1_) were dispersed in a 25 mL ethanol–water solution (1 : 1 v/v) and heated to a specific temperature (*X*_2_) using an ultrasonic bath (USC 300T, VWR, UK) operating at 45 kHz for a predetermined duration (*X*_3_). Subsequently, the mixtures were separated by centrifugation (ORVALL ST 8R, Thermo-Fisher, UK) at 3900 rpm for 40 min. The supernatant solution was then collected, and ethanol was evaporated in a rotary evaporator RV-8-Flex (IKA, Germany) at 170 rpm and 45 °C until the initial volume was reduced to ∼5 mL. The extract was further processed through Modulyo freeze-drying (Edwards, UK).

#### 2.2.4. Alkaline pectin extraction from CPH powder

The process was conducted by modifying a methodology proposed in the literature.^13,14^ A known amount of CPH powder (*X*_1_) was dissolved in a glass bottle containing 150 mL of 0.05 M NaOH and heated to a specific temperature (*X*_2_) using an ultrasonic bath for a predetermined duration (*X*_3_). Subsequently, phase separation of cellulose and pectin was achieved through centrifugation at 3900 rpm for 60 min. The supernatant was collected and combined with 96% ethanol in a 1 : 1 (v/v) ratio, followed by storage at 4 °C for 24 h to precipitate the pectin. After refrigeration, the mixture was centrifuged at 2000 rpm for 30 min to recover pectin in pellet form. The obtained pectin was then placed on non-stick baking paper and dried in an incubator MIR-154 (Panasonic, Japan) at 37 °C until a constant weight was achieved. Finally, the pectin flakes were collected and stored for further analyses.

#### 2.2.5. Enzymatic pectin extraction from CPH powder

The extraction was conducted using a cellulase enzyme, in accordance with the recommended enzyme load (*X*_1_) provided by the manufacturer (Table 1). A known quantity of CPH powder (*X*_2_) was dispersed in a conical flask containing 25 mL of sodium acetate buffer with a pH of ∼5.2 and placed in a shaking incubator 230 VAC (INFORS HT, Switzerland) operating at 175 rpm and 50 °C for a specified duration (*X*_3_). After the catalysis, the enzyme was deactivated at 85 °C for 10 min in an ultrasonic bath; then, the process continued as described in the alkaline extraction method mentioned above.

### 2.3 LbL-MNP manufacturing

CH as a polycation and PEC as a polyanion were used as polyelectrolytes (PEs) for the manufacturing of the LbL-MNPs. Briefly, two different solutions (1 mg mL^−1^) were prepared by dissolving both PEs in dH_2_O and CH in a 1% v/v glacial acetic acid solution. The pH of both solutions was adjusted using NaOH (0.25 M) and HCl (0.1 M) to reach a final value of 6. The negatively charged extracted metabolite mix was added to the PEC solution at a 1 : 4 weight ratio. The LbL deposition began by immersing 20 mg of the negatively charged MNPs into 10 mL of the polycationic CH solution and sonicating in an ultrasonic water bath for 2 min until the solution became homogeneous. Then, after 15 min of shaking at 95 rpm using an Orbital shaker SSM1 (Cole Parmer/Stuart), the MNP solution was centrifuged at 4400 rpm for 5 min. Following this, two washing steps were performed by replacing the polyelectrolyte solution with 5 mL of dH_2_O buffer solution at pH 6, followed by centrifugation (5 min at 4400 rpm) after 10 min of shaking at 95 rpm. Before the final centrifugation step, 100 μL of LbL-MNPs were collected for *ζ*-potential analysis. After completing the washing steps, the buffer solution was removed, and 10 mL of the polyanionic PE was added for the deposition of the second layer following the same procedure described before. The immersion into the polyelectrolyte solutions was repeated until five oppositely charged layers were consecutively deposited. The LbL-MNP systems were freeze-dried and then, stored in a vacuum desiccator (SLS, UK) for further testing.

## 3. Characterisation

### 3.1 Physico-chemical characterisation of the CPH extracts

#### 3.1.1. Determination of sugar content

The sugar content in the extracted pectin samples was determined by adapting established protocols^15,16^ in two stages: (1) polysaccharide hydrolysis: exactly 1 mg of pectin was accurately weighed and placed in a sealed glass vial. Distilled water (1000 μL) and concentrated trifluoroacetic acid (500 μL) were added to achieve a 4 M concentration. After sealing the vial, the mixture was heated with continuous stirring at 120 °C for 90 min and then cooled down. Subsequently, 200 μL of methanol was introduced, and the sample was evaporated to dryness using nitrogen and a heating block (80 °C) to remove trifluoroacetic acid. Finally, the dried hydrolysed pectin was dissolved in 1 mL of dH_2_O; (2) monosaccharide derivatisation using 1-phenyl-3-methyl-5-pyrazolone (PMP): 200 μL of the hydrolysed pectin solution was mixed with 80 μL of 0.3 M NaOH and 80 μL of PMP in methanol. The mixture was incubated at 80 °C for 60 min, and then cooled down and neutralised by adding 80 μL of 0.3 M HCl. Finally, the sample was diluted to 1 mL by adding 400 μL of water. Excess PMP was removed by partitioning with CH_2_Cl_2_ (3 × 1 mL), and phase separation was achieved in a vortex FB15012 TopMix (Fisher Scientific, UK) at 30 000 rpm for 30 seconds. All sugar standards underwent the same hydrolysis and derivatisation conditions as the samples. Prior to chromatographic analysis, all samples were filtered through 0.45 μm hydrophilic nylon syringe filters.

Sugar monosaccharides in pectin samples were quantified using UV at 250 nm through chromatography using an ACQITY Premier UHPLC system, equipped with a single quadrupole mass spectrometric detector. The separation process employed a C18 Column ACQUITY UPLC (50 × 2.1 mm, 1.7 μm) operating at 25 °C. The mobile phase consisted of (A) 20 mM ammonium bicarbonate/ammonium hydroxide (pH 9.2) combined with acetonitrile (85 : 15 v/v) and (B) pure acetonitrile. Upon injecting 5 μL of the PMP-derivatised sample, elution occurred at a flow rate of 0.3 mL min^−1^, following a gradient of (% B in A): (0–5%) 0–8 min, (5–10%) 8–12 min, (10–20%) 12–15 min, (20–0%) 15–16 min, and isocratic conditions for 16–17 min. Peak identification was confirmed through co-chromatography with authentic samples and analysis of molecular masses using ESI-MS with positive polarity. The mass range covered 400–1000 *m*/*z* with a scan time of 200 ms. The capillary voltage was set at 0.8 kV, the covoltage at 30 V, and the gas temperature at 600 °C. Pseudo-molecular ions of tagged pentoses were detected at 481 *m*/*z*, deoxy-hexoses at 495 *m*/*z*, hexoses at 511 *m*/*z*, and uronic acids at 525 *m*/*z*.

#### 3.1.2. Determination of the esterification degree

The esterification degree (DE) was determined by measuring the absorbance of redissolved pectin samples using Fourier transform infrared spectroscopy (FTIR-ATR) (par. 3.1.4). The redissolved pectin was produced by suspending approximately 10 mg of dry pectin from each extraction method (alkaline and enzymatic) in 5 mL of dH_2_O. The pH of the solution was then adjusted to ∼5.5 to ensure that it was above the p*K*_a_ of polygalacturonic acid (3.38), where all non-esterified carboxylic groups in the pectin solution exist as carboxylate ions.^17^ After drying, the readjusted pectin spectra were recorded. Signal processing involved baseline correction and second derivative analysis. The area of peaks in the regions of 1780–1720 cm^−1^ related to (COOH) and 1640–1600 cm^−1^ to COO^−^ was used to calculate the esterification degree based on the following equation:
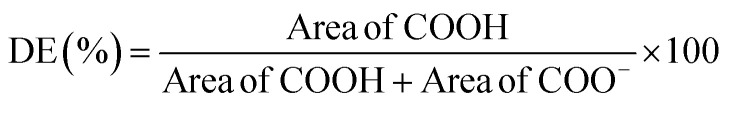


#### 3.1.3. Metabolomics

The material (10 mg) was extracted in 1 mL of analytical methanol. The samples were sonicated for 15 min in an ice-water bath and then centrifuged at 15 000 rpm for 15 min at 4 °C. The supernatants were collected and dried in a vacuum pre-concentrator at 45 °C for 2 h. The dried extracts were, then, resuspended in 100 μL of 95/5 (LC/MS-grade water/acetonitrile), sonicated for 15 min, and passed through a 0.22-micron Costar Spin X filter at 10 000 rpm for 5 min. The resulting filtrate was transferred to a 1.5 autosampler vial with a 200 μL micro-insert. Two extraction blanks and a pooled quality control (QC) were generated as part of the extraction protocol for the data-dependent analysis template (extraction blanks for the MS/MS exclusion list and pooled QC for the inclusion list and analytical stability assessment, respectively).

Chromatographic separation was performed using a Waters High-Strength Silica T3 liquid chromatography column (2.1 mm ID, 150 mm, 1.7 micron) operating at 35 °C with a flow rate of 250 μL min^−1^. A binary buffer system was set up with negative mode buffer 0.1% (ammonium hydroxide). The injection-to-injection time was 21.5 min. Buffer A (95/5 LC/MS-grade water/acetonitrile) and Buffer B (5/95 LC/MS-grade water/acetonitrile) were used in a gradient: T0 95% (A), T1.5 min 95% (A), T11.5 min 5% (A), T15 min 95% (A), and T20 min 95% (A). The heated electrospray ionisation parameter was set at 2.5 kV, with a vaporiser temperature of 275 °C, an ion transfer tube temperature of 300 °C, and gas flow rates as follows: sheath gas (35), aux gas (7), and sweep gas (0). Both data sets were acquired using the data-dependent analysis methodology with the Thermo AcquireX work package. MS1 had a 30 000 mass resolution, mass range of 100 *m*/*z*–1000 *m*/*z*, Quad isolation RF frequency (30), and Automatic Gain Control (AGC) custom mode: 95% with a maximum injection time of 50 ms. MS2 had a 30 000 mass resolution, stepped energy at 10, 35, and 60 HCD, and an isolation width of 1.0 *m*/*z*. Extraction blanks were used for the exclusion list generation. Data sets were converted to .abf file format and aligned using the Riken MSDial 4.9 Metabolite workflow. MS2 matching was performed using an authentic compound library. MS/MS similarity matches of 70% or higher (forward and reverse MS spectrum matching average) with a 10 ppm tolerance for both MS1 and MS2 were considered. QC variation during data acquisition metrics for negative (1.3%) mode had a maximum threshold of 15%. Only MS/MS signals stable within the QC responses with a 25% RSD or lower were retained, along with the corresponding chemical formula, signal-to-noise ratio, retention time and *m*/*z*, adduct, MS/MS match score, and the international chemical identifier ID (InChIKey).

#### 3.1.4. Fourier transform infrared spectroscopy analysis

The functional groups of CPH powder and extracted pectin were identified through FTIR-ATR analysis of the samples, using a Frontier ATR-FTIR instrument (PerkinElmer Inc., UK). Samples were directly placed on a universal ATR sampling accessory and the spectra were recorded in the wavelength range of 4000–550 cm^−1^. Each spectrum resulted from averaging 32 scans with a resolution of 2 cm^−1^. Then, the spectra were pre-processed to compensate for and remove bias linked to experimental evaluation by baseline correction and multiplicative dispersion correction with R Core Team statistical software using the ChemoSpec package (3.6.3, R statistics, USA).

#### 3.1.5. Antioxidant capacity

Different methods were employed to assess the antioxidant capacity of the extracted compounds, including Total Phenol Content (TPC), FRAP and DPPH assays. The pectin antioxidant capacity is presented as normalised by the weight of the sample, previously dried in a SVAC1-2 vacuum oven (Shel-Lab, UK) for 24 h; at least four measurements were recorded for each antioxidant assay.

##### 3.1.5.1. Total phenol content

The TPC of the extracts was determined using the Folin–Ciocalteu method.^18^ Briefly, 10 mg of dry pectin were suspended in 1 mL of dH_2_O, and then 50 μL of the resulting extracts were mixed with 430 μL of dH_2_O and 20 μL of the Folin–Ciocalteu reagent. After stirring, a 20% w/v Na_2_CO_3_ solution was added and allowed to stand for 10 min. The mixture was then diluted with 450 μL of dH_2_O, and its absorbance was measured at 680 nm using a UV-vis spectrophotometer FLUOstar Omega (BMG Labtech, Germany), against a blank prepared with 50 μL of ultrapure water. A calibration curve was generated using gallic acid dissolved in dH_2_O at seven known concentrations ranging from 0.05 to 5 mg g^−1^ (*R*^2^ = 0.998).

##### 3.1.5.2. Ferric reducing antioxidant power

FRAP determination was performed on the optimised extracts and pectin using an Invitrogen™ assay kit from Thermo Fisher Scientific, UK. The necessary solutions for the assay, including 1× buffer and colour solution (FRAP reagents A and B), were prepared according to the manufacturer's specifications. Samples were dissolved in dH_2_O at a concentration of 1 mg mL^−1^ and then diluted at a 1 : 10 ratio in 1× buffer. Then, 20 μL of the prepared samples were added to the appropriate wells with 75 μL of the colour solution. After incubation for 30 min at room temperature, the absorbance of the resulting-coloured product was measured at a wavelength of 560 nm using a FLUOstar Omega MicroPlate Reader. A calibration curve was generated using ascorbic acid dissolved in dH_2_O at six known concentrations ranging from 0.05 to 1000 μM (*R*^2^ = 0.999).

##### 3.1.5.3. 2,2-Diphenyl-1-picrylhydrazyl

The DPPH method was performed following the method proposed by Brad-Williams *et al.*^19^ A methanolic DPPH solution containing 14.5 mg in 500 mL was prepared. Hydrolytic extracts were obtained by stirring ∼100 mg of pectin and 10 mL of ethanol–water 1 : 1 (v/v) solution for 1 h, followed by paper filtration. Each sample was taken in an amount of 100 μL, and 1.95 mL of the DPPH solution was added, performing this procedure in duplicate. In addition, as a control, 1000 μL of 80% (v/v) methanol and 1.95 mL of the DPPH solution (duplicate) were added to a test tube. All tubes were shaken and, then, incubated in the dark for 30 min. The absorbance of the resulting product was measured at a wavelength of 517 nm using a FLUOstar Omega MicroPlate Reader. A Trolox calibration curve (*R*^2^ = 0.998) was constructed from a 10 μmol mL^−1^ stock solution in the range of 50–800 μmol mL^−1^ Trolox.

#### 3.1.6. Determination of theobromine and caffeine

Caffeine and theobromine determination was carried out using an HPLC-DAD 1260 Infinity II (Agilent Technologies, USA), following the method proposed by Rodriguez *et al.*^20^ An extract of ∼200 mg of pectin was dissolved in 5 mL of type I distilled water. Then, 0.75 mL of Carrez I and II solutions were added to each sample in 100 mL of dH_2_O. The samples were centrifuged at 7000×*g* for 10 min in a Bioprocen 22 R centrifuge. The supernatant was collected and filtered through 0.22 μm nylon membranes for HPLC-DAD analysis. A mobile phase of acidified water with 1% glacial acetic acid (A), pH 3.1 (v/v) (A), and acetonitrile (B), with an injection volume of 5 μL, flow rate of 1 mL min^−1^ and a C18 column (5 μm × 4.6 mm id × l : 150 mm) were used. A calibration for both analyte curves was established in the range of 10 to 120 ppm, and the analytes were detected at 280 nm (*R*^2^ = 0.998).

### 3.2 LbL-MNP physico-chemical and morphological characterisation

#### 3.2.1. Determination of *ζ*-potential through dynamic light scattering

Dynamic light scattering was employed to measure the *ζ*-potential of polyelectrolyte solutions and the surface charge of MNPs before and after functionalisation *via* LbL assembly using a Zetasizer Nano-S90 (Malvern Instruments, UK). For each solution, 100 μL aliquots were gently mixed with 900 μL of dH_2_O and introduced into the Zetasizer cuvette. All measurements were taken in triplicate, and the results were expressed as mean ± standard deviation after calculating the mean values.

#### 3.2.2. Fourier transform infrared spectroscopy analysis

The surface modification of the LbL-MNPs was analysed using a Frontier ATR-FTIR instrument, following the method mentioned earlier for the determination of the degree of esterification.

#### 3.2.3. X-ray photoelectron spectroscopy analysis (XPS)

LbL-MNPs underwent XPS analysis to quantitatively evaluate the elemental composition of the biomaterial surface. The examination was conducted using a scanning microprobe Kratos Axis UltraDLD XPS spectrometer (Harwell XPS Service, UK), equipped with a monochromatised AlKα X-ray radiation source. The base pressure in the analysis chamber was 10^−9^ mbar. High 96 power mode was employed for analysis with an X-ray take-off angle of 45°, and a scanned size of ∼1400 × 200 μm. Each specimen underwent survey scans (fixed analyser transmission mode, binding energy range 0–1200 eV, pass energy 117.4 eV) and high-resolution spectra (FAT mode, pass energy 29.35 eV) for C 1s and O 1s. Atomic concentration (At.%) on the survey scan was determined using the built-in CasaXPS software package. To identify the binding energy representing the chemical binding states of each element within the films, XPS spectra for the detected chemical elements underwent peak deconvolution.

#### 3.2.4. Morphology characterisation through a transmission electron microscope (TEM)

A transmission electron microscope (Philips CM 100 Compustage FEI) operating at 100 kV was employed to determine the morphology of LbL-MNPs. For sample preparation, a 10 μL aliquot of the supernatant, extracted before the final centrifugation, was directly added to the TEM grid and positioned in the specialised chamber at the microscope's centre. Diameter measurements were conducted using ImageJ software.

#### 3.2.5. Antioxidant capacity

The antioxidant capacity of the LbL-MNPs was determined according to the Folin–Ciocalteu method and the FRAP assay as described before.

#### 3.2.6. *In vitro* biological analysis

Neonatal Normal Human Dermal Fibroblasts (NHDFs) were obtained from Lonza Biosciences (Switzerland) and cultured following the supplier's recommendations. The fibroblasts were incubated at 37 °C with 5% CO_2_ in Dulbecco's Modified Eagle Medium (DMEM, Merck, UK), supplemented with 10% fetal bovine serum (FBS), 2 mM l-glutamine, and a 1% antibiotic mixture of penicillin and streptomycin (100 U mL^−1^). For biocompatibility assessments, various samples of the metabolite mix and LbL-MNP solutions were prepared at different concentrations (10, 50, 100, 250, 500, and 1000 μg mL^−1^) in a 48-multiwell plate. The solutions were dissolved in DMEM and sterilised by filtration using a 0.22 μm Millex GP PES membrane syringe-driven filter unit (Millipore, SLS, UK) using 5 mL plastic syringes. Suspensions containing 10 000 cells in DMEM were seeded in 96-well plates overnight before the addition of the sample solutions and kept incubated for 48 hours.

To assess the cell viability, a two-colour fluorescence assay known as Live/Dead assay (LIVE/DEAD® Cell Imaging Kit, Life Technologies, Thermo Fisher Scientific, UK) was used following the manufacturer's instructions. Briefly, the L/D staining solution was prepared by mixing 1 μL of calcein with 4 μL of ethidium in 2 mL of PBS. After the incubation period, all wells were washed twice with PBS and incubated with 100 μL of the L/D staining solution for 30 min at 37 °C. Finally, the samples were imaged using an EVOS M5000 fluorescence microscope (Thermo Fisher Scientific, UK).

To assess the cell metabolic activity, PrestoBlue™ assay (Thermo Scientific, UK) was used. After a 48-hour incubation, the culture medium was removed, and the samples were washed with pre-warmed PBS at 37 °C. The PrestoBlue reagent, pre-warmed at 37 °C, was diluted in DMEM at a 1 : 10 ratio. The vial containing the reagent was covered with aluminium to protect the solution from light. Subsequently, 200 μL of the reagent solution was added to each well and incubated for 1 hour at 37 °C with 5% CO_2_. After the incubation, 100 μL of each well's solution was transferred to a white-bottom 96-well plate, and a FLUOstar Omega MicroPlate Reader was used to measure fluorescence (560 nm excitation and 590 nm emission). The obtained values were corrected by subtracting the average fluorescence of control wells containing only PrestoBlue solution. The results are presented as viability (%), determined by comparing the fluorescence value of each sample with the average fluorescence value of the control cells, which were incubated with only DMEM.

To qualitatively characterise H_2_O_2_-induced intracellular ROS generation and assess the antioxidant behaviour of extracts and functionalised nanoparticles, the 2′,7′-dichlorofluorescin diacetate (DCFDA) fluorescence assay was employed. Various samples of the metabolite mix and LbL-MNPs were prepared at different concentrations (10, 50, 100, and 500 μg mL^−1^), including MNPs purchased from Merck (UK) and a solution of resveratrol used as negative and positive controls, respectively. NHDFs were then seeded in a 96-well plate (10 000 cells per well) and incubated with 100 μL of each sample for 24 hours at 37 °C with 5% CO_2_. Upon completing the incubation period, the medium was removed, and each well was washed with PBS pre-warmed at 37 °C. Subsequently, 50 μL of a 150 μM H_2_O_2_ solution was added to each well and left in an incubator for 1 h at 37 °C with 5% CO_2_. The final step involved adding 50 μL of a 10 μM DCFDA solution to each well and incubating for an additional 30 min at 37 °C with 5% CO_2_. After this incubation, 100 μL of each well's solution was transferred to a white-bottom 96-well plate, and a FLUOstar Omega MicroPlate Reader was utilised to measure fluorescence (488 nm excitation wavelength and 520 nm emission wavelength). The results are expressed as ROS generation (%) obtained by comparing the fluorescence value of each sample with the average fluorescence value of the control cells (incubated with only DMEM and treated with H_2_O_2_ solution). Additionally, images of the 96-well plate were captured using an EVOS M5000 fluorescence microscope.

### 3.3. Statistical analysis

The results were presented as means ± standard deviations. Statistical analysis was carried out using GraphPad Prism Software (version 8.4.1). Initially, a one-way ANOVA with repeated measurements was employed. Subsequently, a Tukey's post hoc test was conducted to identify the main factors contributing to data variability. The level of statistical significance was set as follows: * for *p* < 0.05, ** for *p* < 0.01, and *** for *p* < 0.0001. The infrared data were analysed using principal component analysis (PCA) to reduce the dimensionality of the spectral data (15 samples × 900 wavenumbers of MIR-spectra) and to identify patterns and relationships in the data. Prior to analysis, the matrix was scaled and centred, to ensure a matrix mean of zero and a standard deviation of one. This analysis was performed with R Core Team statistical software (3.6.3, R Statistics, USA).

## 4. Results and discussion

### 4.1. Optimisation of the extraction of valuable biocompounds from CPHs and their physico-chemical characterisation

**Fig. 1:**
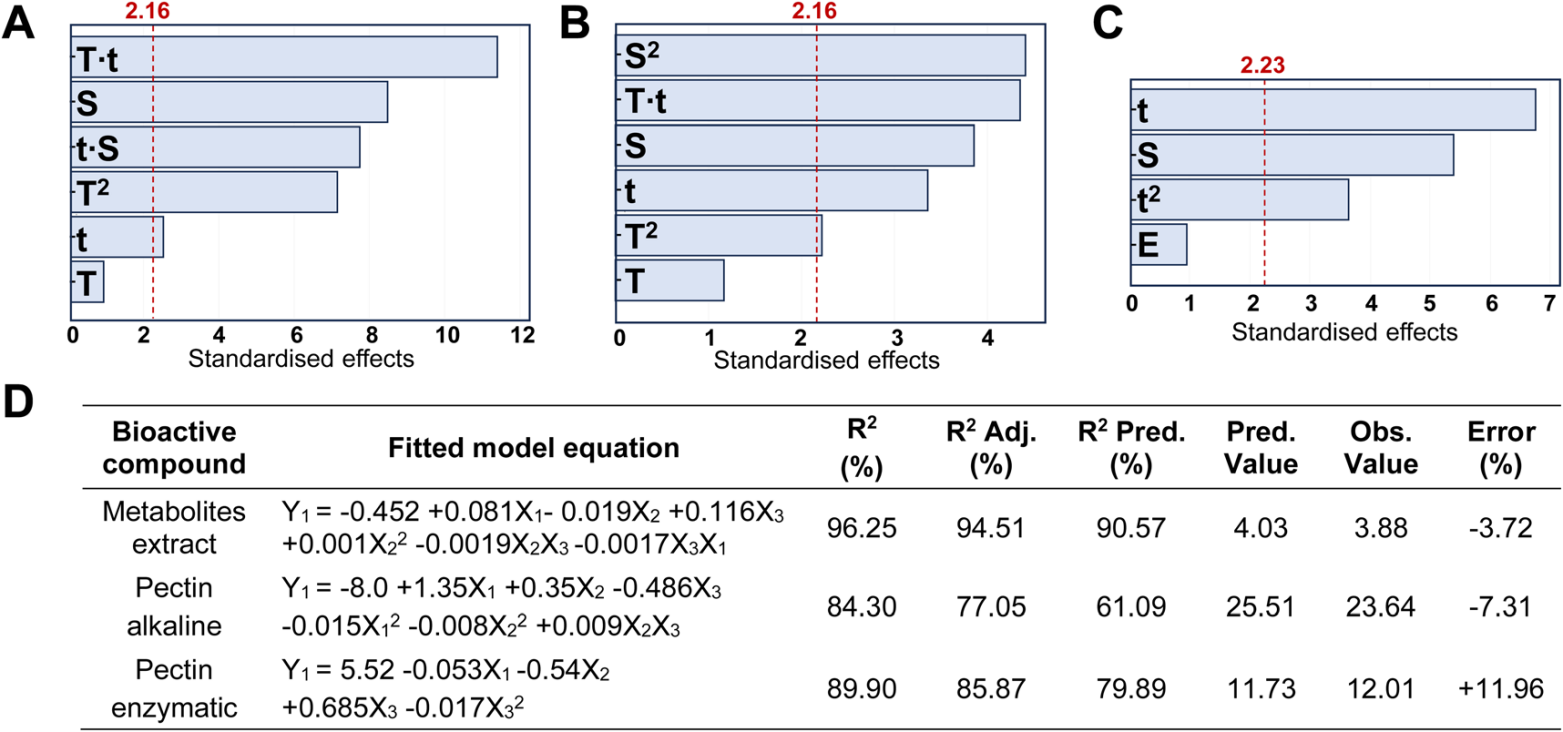
Pareto charts of the standardised effects of the factors (*T*: temperature, *t*: extraction time, *S*: solvent/CPH ratio, and *E*: enzyme/CPH ratio%) and their interactions on the response variable: (A) TPC (GAE, mg g^−1^) in the metabolite mix, (B) extraction yield (%) of pectin in the alkaline route, and (C) extraction yield (%) of pectin in the enzymatic route. The bars, representing the factors, that cross the red dashed line are considered significant (*α* = 0.05); (D) fitted model equations calculated using Minitab software and metrics including the coefficient of determination (*R*^2^), adjusted coefficient of determination (*R*^2^ Adj.), predictive coefficient of determination (*R*^2^ Pred.), the predicted value of extraction yield from the fitted model (Pred. Value), the experimental value of extraction yield (Obs. Value) and the experimental percentage error (Error) calculated as the ratio of the absolute difference between the observed and predicted values and the observed value (%).

Different extraction processes were used and compared to find the optimal procedure to valorise CPHs in terms of yield and quality of the extracted biocompounds. Fig. 1A–C show the Pareto charts illustrating the standardised effects of the factors and their interactions, ranking their significance in the extraction processes, arranged from the largest to the smallest effect. Additionally, the charts include a reference red dashed line. When bars cross this reference value, it indicates that the factors exert a statistically significant influence on the extraction process. In our work, the response variable for the metabolite mix was the TPC, whereas for pectin the extraction yield was considered. It was observed that the CPH powder amount in relation to the extraction solvent volume (indicated with S) significantly influenced all the investigated routes. Notably for the alkaline-extracted pectin, this parameter was optimised as 44.6 mL g^−1^ (Table S2†) without reaching the upper limit (50 mL g^−1^) of the factor (*X*_1_) in the DoE. Instead, for the enzymatic-extracted pectin and metabolite mix, it did attain the lower and upper limits, respectively (Tables S1 and S3†). In addition, time significantly influenced all three extraction procedures, resulting in enhanced performance of the response variable with longer durations when extracting pectin. Also, it was observed that an increase in the enzyme/material weight ratio did not substantially enhance the pectin extraction yield. This behaviour has also been reported by other authors in the extraction of pectin from artichoke by-products.^13^

Furthermore, the optimisation of the metabolite mix, based on the combination of the shortest extraction time (∼16 min) with the highest extraction solvent-to-solid ratio (35.11 mL g^−1^), yielded an observed TPC value of 3.88 ± 0.06 GAE (mg g^−1^) *versus* a predicted value of 4.03 GAE (mg g^−1^) (Fig. 1D). Also, in this process, all correlation coefficients exceeded 90%, which may be attributed to the simplicity of the extraction process, indicating a strong correlation between the response and independent variables.^21,22^ The percent error for this extraction, calculated based on the predicted and observed values, was less than 4%. Our results for the antioxidant properties are comparable to other studies conducted in cocoa shells, presenting values in the range of 2–7 mg g^−1^ of GAE.^23^ When the optimisation was achieved, the temperature did not appear to have a significant effect; however, when considering this factor in the model, it was recommended to conduct extractions at a maximum temperature of 56.8 °C. Note that polyphenols can withstand temperatures in the range of 100 to 150 °C; however, previous work reported that when a higher temperature is used, a short processing time is required.^24^ Moreover, the optimised metabolite mix showed an antioxidant capacity of 996.7 ± 8.06 AAE (μM), evaluated *via* FRAP assay, presenting similar values of CPHs extracted from roasted cocoa beans at different temperatures.^25^

**Fig. 2:**
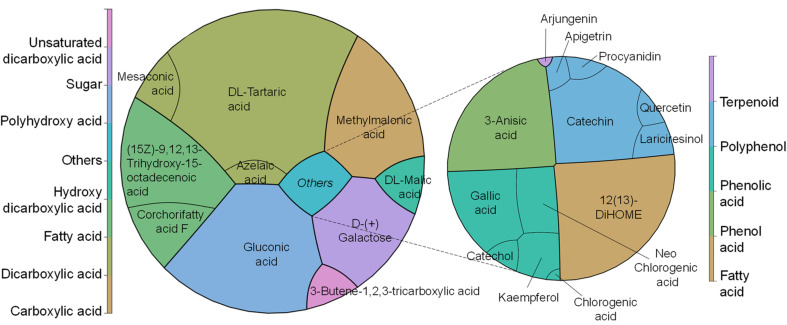
Major metabolic components identified (left) in the CPH metabolite mix with (right) a focus on the compounds with antioxidant and anti-inflammatory properties.

In our study, we have also investigated the properties of the metabolite mix by metabolic analysis that identified 124 compounds (Table S4†), with an MS/MS score of 70% or higher for both CPH powder and ethanol–water extract. In both cases, the first 32 metabolites (Q1) represented ≥96% of the relative abundance of the samples. Coincidentally, the first 9 compounds were the same in both samples but presented variations in their proportions after extraction (Fig. 2). Particularly, in the metabolite mix the major compound was tartaric acid (∼27.5%), a naturally occurring organic acid found in various fruits.^26^ Like other organic acids, it possesses antioxidant properties that may help counteract oxidative stress induced by hydroxyl radicals. Additionally, the second most abundant compound was gluconic acid, a mild organic acid derived from glucose by a simple oxidation reaction.^27,28^

Both tartaric acid and gluconic acid (that could potentially be present in its salt form sodium gluconate) are considered to have high sequestering power, acting as a reducing agent. As described later, the FRAP assay is based on the reduction of iron ions, and so these metabolites with reducing properties could enhance the assay results by contributing to the reduction of iron ions. Also, galactose, probably as a residue from pectin degradation as shown in our sugar composition analysis, and methylmalonic acid, an organic acid, were detected. The latter one at elevated levels in blood or urine may indicate certain metabolic disorders, such as vitamin B12 deficiency.^29^ Other compounds of interest found in the extract were corchorifatty acid F and l-malic acid. As reported by Souza *et al.*,^30^ these moieties, identified in *K. daigremontiana* plants, have potential as bioactive agents, shedding light on their safety and pharmacological potential. It is noteworthy that the detected mesaconic acid also has excellent antioxidant properties with a strong ability to eliminate hydroxyl radicals.^31^ Finally, the metabolic analysis showed the presence of known antioxidants, such as gallic acid, catechin, kaempferol, quercetin and anti-inflammatory compounds, such as chlorogenic acids, apigetrin, and arjungenin, that account for 3.4% of the metabolite mix.

Concerning the extracted pectin, under optimised conditions, the alkaline route achieved a higher yield, reaching 23.6 ± 0.2% (Table S2†) compared to the enzymatic method, which yielded 13.1 ± 0.1% (Table S3†). Notably, these results from the alkaline pectin extraction are consistent with those reported by Cui *et al.*,^32^ who extracted pectin from grapefruit peel. They obtained yields in the range of 21–25% using different NaOH solutions at pH values ranging from 10 to 11. Additionally, our extraction yield exceeded that of many acidic extraction conditions applied to CPHs, typically falling within the range of 1.3–15%. Furthermore, our enzymatic extraction results were equal to or greater than those reported in the literature for CPH pectin extracted using enzymatic methods, with reported extraction yields reaching up to ∼13%.^2^ For both alkaline and enzymatic extractions, the *R*^2^ coefficients were above 80% and the prediction coefficients (*R*^2^ Pred.) ranged from 80 to 60%, respectively. The results are comparable with those of other studies conducted with similar approaches reporting coefficients in the same range; for example, Pinto *et al.*^21^ reported *R*^2^ predicted values >56% when extracting phenolic antioxidants from nut shells using subcritical water. Moreover, the percent error for both pectin extractions was less than 12%, consistent with findings from other studies that extracted pectin under acidic conditions.^33^ Certainly, reducing experimental errors could facilitate technology transfer aimed at exploiting the extraction of valuable components from any biomass waste.

Also, we investigated the phenolic compound content and antioxidant capacity of the extracted pectin, owing to a significant rise in interest in natural antioxidant agents to be used in functional biomedical, pharmaceutical and food products.^34^ Significant differences (*p* < 0.05) were observed in all assays among the alkaline-extracted, enzymatic-extracted and commercial pectin, with the latter consistently exhibiting lower performance in all three determinations (Table 2). Specifically, the alkaline-extracted pectin exhibited the highest TPC. Together with the enzymatic-extracted pectin, it presented approximately 8 and 5 times more phenols, respectively, than the control. The assessment of antioxidant capacity using the FRAP method revealed significantly superior results for the CPH extracted pectin compared to the control, with similar outcomes observed for both extraction methods in reducing Fe^3+^ ions. Conversely, the enzymatic-extracted pectin displayed the best values in the DPPH assay, demonstrating ∼16% higher ability to scavenge free radicals compared to the alkaline-extracted pectin and ∼67% compared to the commercial citrus pectin. These results are consistent with the findings reported by Djaoud *et al.*,^35^ wherein the assistance of ultrasound enables the extraction of pectin more enriched in antioxidants compared to conventional acidic extractions.

**Table 2:** Total phenol content and antioxidant capacity assessed in extracted and commercial citrus pectin^a^

Assay	Alkaline	Enzymatic	Control
TPC	0.48 ± 0.01^a^	0.30 ± 0.00^b^	0.06 ± 0.06^c^
FRAP	42.19 ± 1.68^a^	39.65 ± 3.59^a^	4.28 ± 2.00^b^
DPPH	0.31 ± 0.02^a^	0.36 ± 0.03^b^	0.12 ± 0.01^c^

a Different letters indicate significant differences (*p* < 0.05) among samples. Results are expressed as TPC in mg gallic acid equivalents per g, FRAP in μM ascorbic acid equivalents, and DPPH in μmol trolox equivalents per g.

Furthermore, a sugar analysis was conducted to determine the structural composition of pectin and understand how the extraction method can yield materials with varying profiles. Table 3 provides information on the pectin structure based on the sugar contents from the monosaccharide composition. It was observed that enzymatic-extracted PEC exhibited a higher content of sugars compared to alkaline-extracted PEC. This method resulted in higher values of uronic acids, ∼78% compared to ∼57% through alkaline extraction; this result indicates a greater linearity of the enzymatic-extracted pectin, as calculated from the ratio between the back-bone sugar GalA and the neutral sugars on side chains (Rha + Ara + Gal),^36^ with values of 1.5 for the alkaline-extracted and 1.9 for the enzymatic-extracted material. Moreover, in the enzymatic route, there was less diversity in terms of sugar monosaccharides; neither xylose nor glucose was detected. This can be attributed to the enzymatic complex, which contains hemicellulase capable of degrading xylose, mannose, glucose, and galactose, common sugars found in hemicellulose.^37^ Furthermore, the PEC side region was presented in a lower proportion for the enzymatic-extracted pectin (∼19%), because this region is susceptible to enzymatic action, making it easily degraded.^38^ In contrast, alkaline-extracted pectin exhibited a total content of arabinose, rhamnose and galactose of ∼29%. Regarding the degree of esterification, both extracted materials were classified as low methoxyl PEC (DE <50%). Indeed, the pH of the extraction solution played a significant role; under optimised extraction conditions, the enzymatic-extracted pectin (pH ∼5) showed a DE of 48.5 ± 4.1%, while with the alkaline route (pH ∼12) it presented significantly lower values (40.1 ± 3.4%). This confirmed that DE values in PEC decreased with an increase in extraction pH.^39^

**Table 3:** Composition of structural sugars (μg per mg sample) of pectin extracted from CPH powder using alkaline and enzymatic methods, measured by UHPLC. Different letters represent significant differences (*p* < 0.05) between the different pectin samples

Route	Pentoses		Deoxy-hexoses		Hexoses		Uronic acids		Total (μg mg^−1^)
	Ara	Xyl	Rha	Gal	Glc	Man	GalA	GlcA	
Alk.	25.2 ± 5.8^a^	9.5 ± 1.8	11.1 ± 1.6^a^	33.5 ± 4.6^a^	8.5 ± 2.6	15.3 ± 7.9^a^	107.7 ± 11.4^a^	30.6 ± 1.7^a^	241.4
Enz.	12.1 ± 9.9^a^	—	3.33 ± 0.9^b^	30.6 ± 0.2^a^	—	9.64 ± 1.3^a^	144.9 ± 5.2^b^	47.5 ± 0.3^b^	248.2

**Fig. 3:**
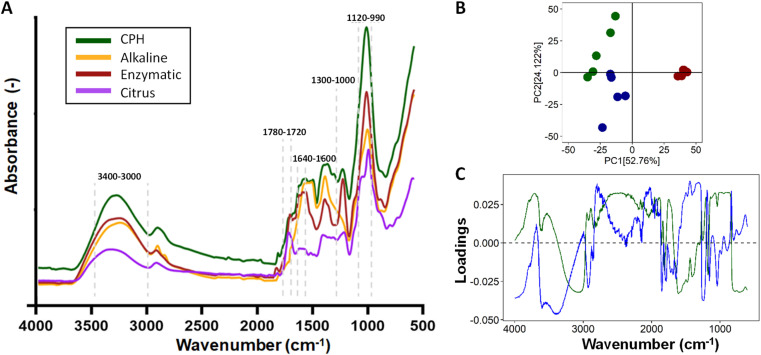
Infrared spectrum analysis of cocoa pod husks and extracted pectin (A), evaluation of pectin quality through Principal Component Analysis (PCA) (B), and an examination of the contribution of each wavelength to the PCA (C).

In addition, the FTIR-ATR spectra of CPHs, commercial pectin, and pectin obtained after alkaline and enzymatic extraction are illustrated in Fig. 3A. Differences in the spectra were evident for each of the samples obtained in the 3400–2800 and 1800–650 cm^−1^ regions. The nature of the individual samples may influence these differences. For example, the concentration of water present in the cocoa husk samples is a critical factor in increasing or decreasing the absorbance of the spectrum. The OH bonds of water molecules have specific vibrations and rotations in regions such as 3400–3000 cm^−1^ and 1639 cm^−1^.^40^ On the other hand, the most critical differences in the vibrations of the spectra of CPHs, commercial pectin, and extracted pectin were evidenced in peaks associated with the degree of esterification (174 3 cm^−1^ and 1631 cm^−1^), gallic acid (1120–990 cm^−1^), and PEC structure cycle (1300–1000 cm^−1^), as well as in the regions of 1780–1720 cm^−1^ related to COOH and 1640–1600 cm^−1^ to COO–.^41^ Notably, the pectin obtained by the alkaline method does not present prominent peaks in the regions of 1780–1720 cm^−1^ and 1640–1600 cm^−1^, which explains its low degree of esterification. The chemometric analysis of the three types of pectin showed a distinct clustering trend for each pectin (Fig. 3B). 76.78% of the explanation of the total variance of the infrared spectrum data shows that the commercial PEC is grouped in the positive part of CP1 (56.72%). In contrast, the PEC samples extracted by the alkaline and enzymatic methods tend to be grouped in quadrants II and III, respectively, showing a high intra-sample variability, which is explained by the heterogeneity of the extraction method. The real loadings plot (Fig. 3C) provided insight into the distribution of the PEC samples, showing that commercial pectin is more influenced by wavenumbers between 850–1150 cm^−1^ and 1700–1780 cm^−1^. The 850–1150 cm^−1^ region could be due to C–O bonds present in PEC sugars.

Conversely, the 1700–1750 cm^−1^ region is associated with C=O stretching bands, *i.e.*, it indicates the presence of carboxyl (COOH) groups.^42^

The PEC with the enzymatic method owes its distribution to a more significant presence in the regions 3000–3400 cm^−1^ and 1800–1600 cm^−1^, and some peaks were present between 1300–1000 cm^−1^. The first region is associated with the bands of OH bond stretching, while the 1800–1600 cm^−1^ region is associated with the vibration of carboxylate (COO–) and ester carbonyl (C=O) groups,^43^ related to uronic acids, which is consistent with a higher content of GalA and GlcA (Table 3) in the PEC obtained by the enzymatic method. The contribution of the peaks evidenced between 1300 and 1000 cm^−1^ may be due to the vibration of C–O–C bonds present in the glycosidic bonds between the sugar monomers, as well as to the vibrations of the bending bands of the C–H bonds in the carbon chain of pectin.^44^ Finally, alkaline-extracted pectin showed a higher ratio in the 1500–1300 cm^−1^ region. In this region, it is possible to find bands associated with the vibrations of the C–H bonds of methyl (–CH_3_) and methylene (–CH_2_) groups in the PEC carbon chains.^45^

Furthermore, theobromine and caffeine were not detected (LOD = 10 ppm) in either the metabolite mix or pectin. The content of these metabolites in CPHs is significantly low, as demonstrated by the metabolic analysis (Fig. 2), eliminating the presence of stimulant products such as caffeine and theobromine in the resulting products. Particularly, caffeine is included in the monitoring program by the WADA/USADA but is not considered a prohibited substance. This result was consistent with previous studies where theobromine and caffeine were absent or in very low concentration in cocoa pod husks compared to cocoa bean shells.^46^

Finally, from the analysis done on the extracted PEC, it could be argued that the alkaline route offers greater advantages in the valorisation of CPHs compared to the enzymatic route. Indeed, the alkaline route yields a larger production of pectin while requiring less time and energy. This contrasts with the enzymatic route, which operates at ∼70 °C for ∼1.5 hours, despite its optimal catalysis temperature of 50 °C for >20 hours. The alkaline extraction time is considerably shorter, representing less than 10% of the time required for enzymatic extraction. Also, complexities related to pricing and storage requirements for enzymes persist as significant obstacles, contributing to increased process costs and making implementation in cocoa-producing countries more challenging.^47^ Additionally, the superior performance of the PEC extracted under alkaline conditions in reducing Fe^3+^, harmful ions known to induce oxidative stress,^48^ played a pivotal role in choosing this material as the polyanion for constructing the LbL-MNPs. These nanoparticles are specifically designed to regulate oxidative stress in cellular experiments.

### 4.2. LbL-MNP physico-chemical characterisation

In this work, the LbL assembly process generated all nanoparticles by utilising the extracted PEC and CH as polyelectrolytes, using mesoporous silica nanoparticles as cores. The experimentally determined *ζ*-potential charges for the building blocks forming the LbL-MNPs were −30.3 ± 2.4 mV for the MNPs, +40.0 ± 2.4 mV for CH and −32.2 ± 2.3 mV for PEC. These findings align with previous reports in the literature, indicating a consistent pattern in LbL assembly when PEC and CH were employed for multi-layered coating fabrication.^49,50^ Also, *ζ*-potential measurements revealed a negative charge for the extracted cocoa metabolite mix (−20.2 ± 4.9 mV), leading to their further incorporation into the PEC solution. Specifically, the metabolite mix was dissolved at a 1 : 4 weight ratio, resulting in a final *ζ*-potential value of −27.8 ± 2.9 mV of the polyanionic solution.

**Fig. 4:**
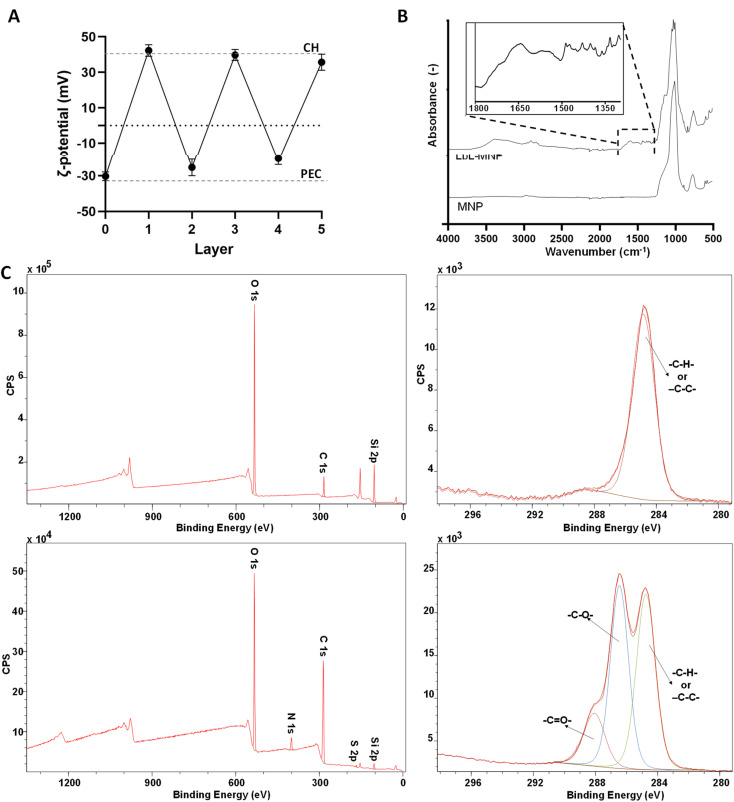
(A) *ζ*-potential measurement as a function of the layer number. The dashed lines represent the saturation values of the CH and PEC layers. (B) FTIR-ATR spectra of the MNPs before and after LbL functionalisation. (C) XPS spectra of the MNPs before (top line) and after LbL functionalisation (bottom line), including survey and deconvoluted C 1s spectra.

The validation of the formation of a 5-layer coated system was confirmed through *ζ*-potential measurements taken after each layer addition (Fig. 4A). In the LbL-MNPs, the *ζ*-potential measurements shifted from an initial value of −30.3 ± 2.4 mV (core of MNPs) to +42.3 ± 3.3 mV after the deposition of the first polycationic layer. Subsequently, for the remaining four layers deposited onto the 1-layer coated nanoparticle precursor, the *ζ*-potential values fluctuated as follows: −25.3 ± 4.7 mV (layer 2), +39.8 ± 3.1 mV (layer 3), −20.2 ± 3.4 mV (layer 4) and +35.7 ± 4.5 mV (layer 5).

The obtained *ζ*-potential values were very close to the corresponding measurements of the starting PE solutions. As illustrated in prior LbL assembly processes,^51^ LbL assembly involves the consecutive deposition of oppositely charged polyelectrolytes, relying on strong electrostatic interactions between polycation and polyanion layers. The resulting *ζ*-potential at each layer indicated the absence of the previously added polyelectrolyte, as corroborated by the uniformity of the peaks in the *ζ*-potential measurement. This surface charge inversion after every deposition step is a fundamental prerequisite for the LbL assembly of PE.

FTIR-ATR analyses were performed on MNPs before and after LbL functionalisation (Fig. 4B). The specific spectrum of MNPs can vary based on the synthesis method, nanoparticle size, and shape.^52^ In this work, we used commercialised MNPs supplied by Merck and characterised by a 200 nm particle size and 4 nm pore size. The MNP spectrum exhibited the most prominent peaks, which clearly depicted the symmetric and anti-symmetric vibrations of *ν*(Si–O–Si) in the 1000–1200 cm^−1^ region and in the 500–800 cm^−1^ region, which aligns closely with the findings of previous studies conducted by Nikolić *et al.*^53^ and De Barros *et al.*^54^ Furthermore, the FTIR-ATR spectrum of the LbL-MNPs revealed a band peak in the region around 3330–3000 cm^−1^, corresponding to –OH stretching, akin to both CH and PEC polyelectrolytes. Additionally, as evidenced in the inset of Fig. 4B, peaks at 1650 cm^−1^ and 1370 cm^−1^ are associated with the C=O stretching vibration of the CH amide carbonyl group and the stretching of the PEC C–O bond, respectively. The LbL-MNPs also displayed peaks at around 1205 cm^−1^, corresponding to C=O stretching vibrations, and absorption bands appeared at 900–600 cm^−1^ due to the stretching vibrations of the entire anhydroglucose ring,^55^ confirming the presence of the CPH-extracted metabolite mix in the multilayered nanocoating.

Moreover, XPS confirmed the successful formation of the multilayer coating on the MNPs once again (Fig. 4C). In the survey spectrum after LbL functionalisation, the N 1s peak at 339.5 eV indicated the successful addition of CH, and the S 2p peak at 167 eV, a characteristic element of the extracted metabolite mix, was observed. The atomic concentration of Si 2p decreased significantly compared to the bare MNPs, dropping from 35.33% to 3.34%. Furthermore, the high-resolution spectra for C 1s, along with the curve fit, exhibit three peaks attributed to different carbon oxidation states: (1) 284.7–285.0, (2) 286.8–287.0, and (3) 288.5–289 eV, corresponding to C–H or C–C bonds, C–O bonds, and N–C=O (amide) groups, respectively.^56^ Specifically, in the bare MNPs only the component at 284.7 eV corresponding to C–C and/or –C–H– bonds was detected, while in the LbL-MNPs the components at 286.9 eV attributed to C–O bonds and at 288.8 eV, indicating the presence of the PE coating, were detected.

**Fig. 5:**
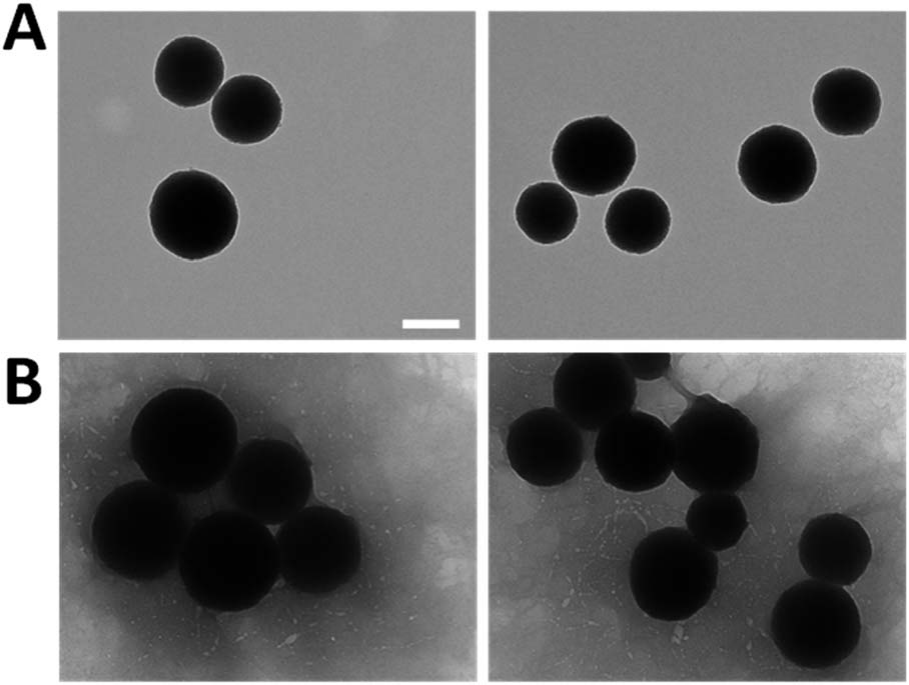
TEM images of bare MNPs (A) and after LbL functionalisation (B). Scale bars = 200 nm.

To enhance the understanding of the obtained results and verify their accuracy, the resulting morphology of the MNPs was examined through TEM analysis as shown in Fig. 5. All the nanoparticles exhibited a spherical and uniform morphology with a slight surface roughness, meeting the requirements typical of this system. Particularly, the size increased from 251.2 ± 27.6 nm of the bare MNPs (Fig. 5A) to 303.2 ± 41.1 nm of LbL-MNPs (Fig. 5B), indicating an approximate 50 nm size increase after the deposition of 5 layers. This means that individual layers contribute less than 10 nm each to the overall size, a finding further supported by Kolman *et al.*^57^ who have investigated the influence of the charge density of mesoporous silica nanoparticles (with an initial size of 20 nm) on the final diameter of these systems functionalised with LbL assembly using polyethyleneimine and carboxymethylcellulose as PEs. They found that after the deposition of 5 nanolayers there was an increase in the diameter reaching a value of 60 nm.

### 4.3. LbL-MNP *in vitro* biological characterisation

**Fig. 6:**
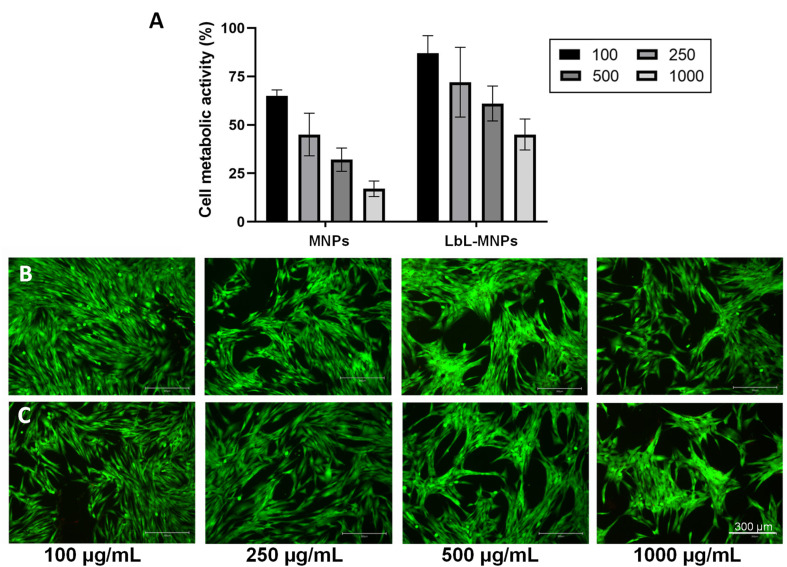
Metabolic activity of neo-dermal human fibroblast cells after 48 h of seeding in the presence of different concentrations (from 100 to 1000 μg mL^−1^) of the LbL-MNPs. Bare MNPs have been used as a control. The results are shown as average ± SD after normalisation to the control of cells seeded on TCPs (A); live/dead images of neo-dermal human fibroblast cells after 48 h of seeding in the presence of different concentrations (from 100 to 1000 μg mL^−1^) of the LbL-MNPs (B) and bare MNPs (C). Scale bar = 300 μm.

Neonatal normal human dermal fibroblasts were seeded with different concentrations of LbL-MNPs (100, 250, 500, and 1000 μg mL^−1^) (Fig. 6A) and their metabolic activity was assessed using PrestoBlue assay after 48 hours. As a control, commercial bare MNPs have also been utilised. Priorly, the cytocompatibility of the metabolite mix was investigated and the results are shown in Fig. S1 in the ESI section† to confirm their cytocompatibility. For the LbL-MNPs, a notable increase in metabolic activity was observed when their concentration was below 250 μg mL^−1^, confirming the findings from the live/dead staining assay (Fig. 6B). Notably, the multilayered coating promoted cell growth and rapid cell spreading in all the samples, with the lowest cell metabolic activity of ∼45% at the highest concentration (1000 μg mL^−1^). Conversely, a significant reduction in cell metabolic activity was observed in cells seeded with the commercial MNPs, with a cell metabolic activity of less than 20% when their highest concentration was incubated. These results align with the literature, emphasising the significance of coating bare MNPs to enhance their cytocompatibility and biodistribution as drug delivery carriers. Interestingly, the different surface modifications can also influence the cell uptake of the MNPs. As an example, the study conducted by Slowing *et al.*^58^ investigated the impact of the surface chemistry of mesoporous silica nanoparticles on their cellular uptake and subsequent endosomal escape. Various MNPs were synthesised by functionalising a mobile crystalline material (MCM-41), the most widely used MNPs, with different groups, including guanidinopropyl (GP), 3-aminopropyl (AP), 3-[*N*-(2-guanidinoethyl) guanidine] propyl (GEGP), and *N*-folate-3-aminopropyl (FAP) groups. All functionalised MNPs exhibited a lower surface area and pore volume compared to the bare MNPs. The results indicated that FAP-MNPs were internalised through both clathrin and folate receptor-mediated endocytosis, leading to a greater confinement of positively charged particles inside endosomes. In contrast, MNPs followed a clathrin-mediated pathway for ingestion, while AP- and GP-grafted MCM-41 MNPs were internalised *via* a caveolae-dependent mechanism. These findings underscore the impact of MNP surface properties, such as surface electrochemistry, on cellular uptake and subsequent endosomal escape.

Moreover, a Live/Dead assay was conducted to qualitatively assess cell morphology after 48 hours of incubation with various concentrations of LbL-MNPs and bare MNPs (Fig. 6B and C). In all samples, cells exhibited a highly elongated and flattened morphology, uniformly spreading across the tissue culture well. However, an apparent decrease in the number of cells was observed with an increase in the concentration of nanoparticles, particularly for the non-functionalised ones. The presence of red dead cells resulted in an inconsistent count compared to viable ones, suggesting low apoptotic activity of bare MNPs and after the LbL functionalisation. This observation could also be attributed to the three washing steps with PBS performed before the Live/Dead assay, which may have washed out some of the dead cells.

**Fig. 7:**
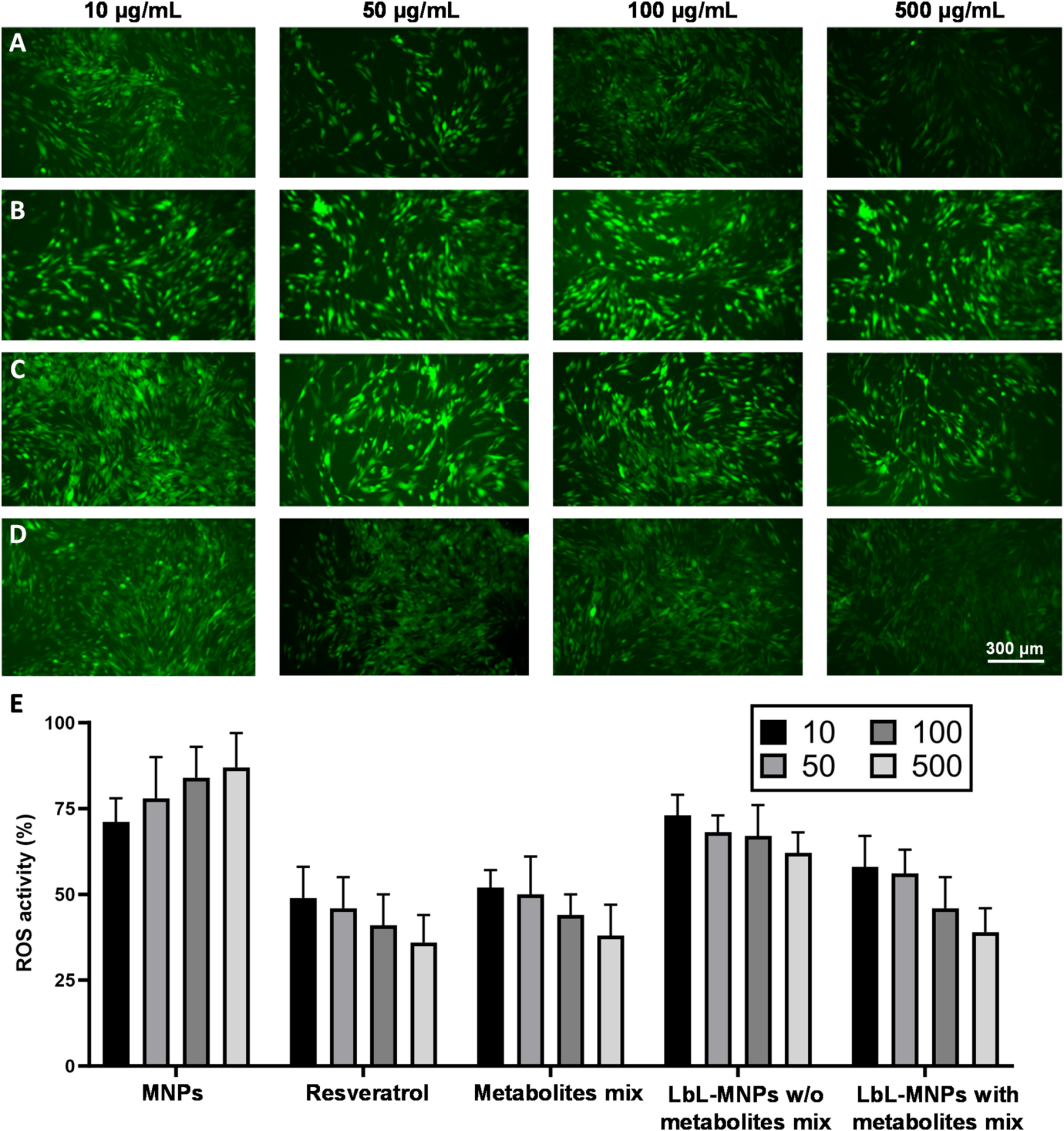
Fluorescence images of the repressive effect of ROS activity under H_2_O_2_-induced intracellular oxidative stress on neo-dermal human fibroblasts, treated with different concentrations (10, 50, 100 and 500 μg mL^−1^) of (A) resveratrol, (B) bare MNPs, and LbL-MNPs without (C) and with (D) the incorporation of the metabolite mix. Bar = 300 μm. (E) Quantification of the ROS activity (%) measured by comparing the fluorescence value of each sample with the average fluorescence value of the control cells.

Finally, cells generate reactive oxygen species (ROS), commonly known as free radicals or oxidants, whose accumulation causes cell damage. Similarly, the accumulation of ROS in skin cells can trigger cellular aging, presenting the primary concern for skin health. In this work, we estimated the properties of the produced LbL-MNPs as antioxidant carriers and DCFDA fluorescence assay was employed to qualitatively characterise H_2_O_2_-induced intracellular ROS generation (Fig. 7), by incubating them in varying concentrations with fibroblast cells for 24 hours. Subsequently, a 150 μM H_2_O_2_ solution was added to each well. Additionally, resveratrol, a naturally occurring polyphenol (*trans*-3,4′,5-trihydroxystilbene) characterised by antioxidant, anti-inflammatory and anti-tumorigenic properties,^59^ was used as a positive control.

The repressive effect of cells incubated with DMEM and treated with H_2_O_2_ (Fig. S2A†), without any treatment (Fig. S2B†) and in the presence of different concentrations of metabolite mix was also investigated (Fig. S2C†). Intracellular ROS generation was examined both under a fluorescence microscope and measured using a microplate reader. As shown in Fig. S2A,† the levels of ROS were significantly higher in H_2_O_2_-exposed fibroblasts, while without the treatment the cells did not show any ROS formation (Fig. S2B†). The measurement of ROS production performed on LbL-MNPs revealed a lower fluorescence intensity with the increase of their concentration, confirming the repressive effect of engineered nanoparticles on H_2_O_2_-induced intracellular oxidative stress (Fig. 7D). In contrast, microscope images of bare MNPs resulted in a higher constant fluorescence intensity for all the sample concentrations tested (Fig. 7B). Furthermore, in Fig. 7A, H_2_O_2_-exposed fibroblasts, treated with varying concentrations of resveratrol solution, demonstrated a weaker fluorescence intensity in samples, where it was more concentrated, confirming its repressive effect on H_2_O_2_-induced intracellular oxidative stress. This has also been reported by Zhuang *et al.*^60^ who found decreased intracellular ROS levels by seeding IPEC-J2 cells with resveratrol under oxidative stress induced by H_2_O_2_. Thus, this positive control demonstrated the successful ability of the manufactured LbL-MNPs to decrease the ROS levels, showing a similar behaviour to the resveratrol positive control. Interestingly, also the LbL-MNPs without the incorporation of the metabolites showed a slight decrease of ROS levels at higher concentration (>100 μg mL^−1^, Fig. 7C) when compared to the bare MNPs. This demonstrated that the extracted pectin is enriched with antioxidant compounds.

Finally, quantitative analysis (Fig. 7E) was also performed on the fluorescence images of all the samples tested. The results demonstrated that ROS levels were significantly higher in bare MNPs, especially at their highest concentration, leading to a ROS activity of 87%. In contrast, the most potent repressive effect on H_2_O_2_-induced intracellular oxidative stress was achieved by free resveratrol and metabolite mix solutions, resulting in ROS activities of 36% and 38%, respectively. Generally, LbL-MNPs also exhibited a significant repressive effect on H_2_O_2_-induced intracellular oxidative stress, especially at higher concentrations, reaching a value of 43%.

It is important to note that the positive controls, which include resveratrol and the metabolite mix, yield an absolute value. In contrast, the fluorescence value obtained for our LbL-MNPs is relative to the weights of the building-block—MNPs, pectin, chitosan, and the metabolite mix—used in forming the nanoparticles. Furthermore, the relationship between ROS reduction activity and the type of sample, as well as its concentration, can depend on various factors. Furthermore, numerous studies have confirmed the correlation between increased oxidative stress and cytotoxicity induced by MNPs.^61^ Petrache Voicu *et al.*^62^ reported a significant rise in ROS production after exposure to MNPs in various cell types and Saehan Choi *et al.*^63^ reported that the generation of ROS was reduced due to the combined effects of the mesoporous silica structure and chitosan coating, due to their antimicrobial and antioxidant properties.

## 5. Conclusions

This study successfully optimised for the first time the extraction processes for valuable biocompounds from CPHs and characterised their physico-chemical properties, aligning with circular economy principles and contributing to the sustainability of the cocoa industry. The investigation focused on extracting a mix of metabolites and pectin—crucial components with applications in nanotherapeutics—and their subsequent application as building blocks. Specifically, the pectin extracted from cocoa pod husks was combined with chitosan to create a nanocoating embedded with the extracted metabolic mix through LbL assembly on MNPs used as a core. The resulting LbL-multifunctionalised nanoparticles showcased a positive impact on fibroblast metabolic activity and morphology, with observed enhancements in cell growth and spreading. Importantly, the manufactured LbL-MNPs exhibited antioxidant behaviour, effectively mitigating H_2_O_2_-induced intracellular oxidative stress, thereby highlighting their potential for biomedical applications. Indeed, these LbL-MNPs can be proposed to be included into electrospun membranes used for skin repair, where their role in decreasing ROS build-up can accelerate wound healing.

## Author contributions

Joel Girón-Hernández and Piergiorgio Gentile: conceived and designed the experiments; performed the experiments; analysed and interpreted the data; contributed reagents, materials, analysis tools or data; wrote and revised the paper. Noemi Corbezzolo, Yeison Barrios Rodríguez, and William Cheung: performed the experiments; analysed and interpreted the data; contributed reagents, materials, analysis tools or data; wrote the paper. Dayana Orozco Blanco and Carlos Carranza Gutiérrez: performed the experiments; analysed and interpreted the data; wrote the paper.

## Conflicts of interest

There are no conflicts to declare.

## Supplementary Material

NA-006-D4NA00248B-s001
