# Supplementary material for: Exploiting residual cocoa biomass to extract advanced materials as building blocks for manufacturing nanoparticles aimed at alleviating formation-induced oxidative stress on human dermal fibroblasts
Source: Nanoscale Adv. 2024 May 30;6(15):3809–24. doi: 10.1039/d4na00248b (PMC11265571; doi:10.1039/d4na00248b)

Electronic Supplementary Material (ESI) for Nanoscale Advances.
This journal is © The Royal Society of Chemistry 2024

Supplementary material

Exploiting residual cocoa biomass to extract advanced materials as building blocks for manufacturing nanoparticles aimed at alleviating formation-induced oxidative stress in cells

Joel Girón-Hernández, Yeison Barrios Rodriguez, Noemi Corbezzolo, Dayana Orozco Blanco, Carlos Carranza Gutierrez, William Cheung, Piergiorgio Gentile

Table S1. Values of explanatory variables: solvent/CPH ratio (X_1), temperature (X_2), extraction time (X_3), and response variable: TPC (Y_1).

Run	X_1, Solvent/CPH ratio (mL/g)	X_2, Temperature (°C)	X_3, Extraction time (min)	Y_1, TPC GA (mg/g)
1	15.0	30.0	50.0	2.95
2	22.5	40.0	37.5	2.50
3	22.5	40.0	37.5	2.50
4	30.0	30.0	50.0	2.85
5	30.0	30.0	25.0	2.68
6	35.1	40.0	37.5	2.81
7	30.0	50.0	25.0	3.02
8	22.5	56.8	37.5	2.80
9	15.0	50.0	25.0	2.55
10	15.0	30.0	25.0	2.06
11	15.0	50.0	50.0	2.41
12	22.5	40.0	16.5	2.41
13	22.5	23.2	37.5	2.78
14	22.5	40.0	37.5	2.46
15	30.0	50.0	50.0	2.34
16	22.5	40.0	37.5	2.52
17	22.5	40.0	37.5	2.40
18	22.5	40.0	58.5	2.59
19	9.9	40.0	37.5	2.27
20	22.5	40.0	37.5	2.41

The optimised conditions for the extraction of antioxidants were as follows: a 35.11 (mL/g) of solvent/ solid ratio (CPH), temperature of 56.8 °C and an extraction time of 16.5 minutes.

Table S2. Values of explanatory variables: solvent/CPH ratio (X_1), temperature (X_2), extraction time (X_3), and response variable: pectin extraction yield (Y_1).

Run	X_1, Solvent/CPH ratio (mL/g)	X_2, Temperature (°C)	X_3, Extraction time (min)	Y_1, Pectin yield (%)
1	50.0	50.0	45.0	18.50
2	40.0	60.0	67.5	20.97
3	40.0	60.0	105.3	20.29
4	56.8	60.0	67.5	17.65
5	30.0	70.0	45.0	16.22
6	30.0	70.0	90.0	14.69
7	40.0	60.0	67.5	18.90
8	40.0	60.0	67.5	18.45
9	50.0	50.0	90.0	15.10
10	40.0	60.0	67.5	20.21
11	23.2	60.0	67.5	11.26
12	40.0	60.0	67.5	19.47
13	40.0	60.0	67.5	17.86
14	40.0	60.0	29.7	15.16
15	50.0	70.0	90.0	22.65
16	30.0	50.0	45.0	16.52
17	40.0	43.2	67.5	15.78
18	30.0	50.0	90.0	17.62
19	50.0	70.0	45.0	15.04
20	40.0	76.8	67.5	17.40

The optimised conditions for alkaline pectin extraction were as follows: ratio of 44.6 mL/g, temperature of 76.8 °C, and an extraction time of 105.3 minutes.

Table S3. Values of explanatory variables: enzyme/ material ratio (X_1), CPH powder concentration (X_2), extraction time (X_3), and response variable: pectin extraction yield (Y_1).

Run	X_1, Solvent/CPH ratio (mL/g)	X_2, g enzyme/ g CPH ratio (%)	X_3, Extraction time (h)	Y_1, Pectin yield (%)
1	50	5.5	6	8.00
2	40	5.5	15	10.31
3	50	1	15	11.40
4	30	1	15	8.69
5	50	10	15	10.40
6	40	1	6	5.33
7	30	10	15	7.26
8	30	5.5	24	8.29
9	40	10	24	9.60
10	30	5.5	6	5.77
11	40	5.5	15	8.98
12	40	10	6	6.13
13	40	1	24	9.87
14	40	5.5	15	8.36
15	50	5.5	24	11.00

The optimised conditions for enzymatic pectin extraction were as follows: a 2% (w/v) of CPH powder dissolved in sodium acetate buffer, a 1% ratio (g enzyme/extraction volume), and an extraction time of 22 hours.

Table S4. List of metabolites identified in CPH metabolites mix with an MS/MS score of 70% or higher, with their relative abundance (%). NP means

Compound	%	Compound	%
DL-Tartaric acid	27.5739	3-Hydroxy-3-(methoxycarbonyl)pentanedioic acid	0.0418
Gluconic acid	16.7958	Kaempferol-7-O-neohesperidoside	0.0391
Methylmalonic acid	11.1273	NP-016455	0.0366
(15Z)-9,12,13-Trihydroxy-15-octadecenoic acid	9.7260	NP-020515	0.0365
D-(+)-Galactose	7.4446	5,7-Dihydroxy-2-(4-hydroxyphenyl)-6,8-bis[3,4,5-trihydroxy-6-(hydroxymethyl)tetrahydro-2H-pyran-2-yl]-4H-chromen-4-one	0.0345
Corchorifatty acid F	3.6382	NP-015948	0.0332
DL-Malic acid	2.1296	4-Hydroxybutyric acid (GHB)	0.0317
Azelaic acid	1.9499	4-oxo-4,5,6,7-tetrahydrobenzo[b]furan-3-carboxylic acid	0.0315
Mesaconic acid	1.2971	4-Acetyl-3-hydroxy-5-methylphenyl β-D-glucopyranoside	0.0313
3-Butene-1,2,3-tricarboxylic acid	1.2112	6-Hydroxypicolinic acid	0.0297
α,α-Trehalose	1.1467	Abscisic acid	0.0296
(2R)-2,3-Dihydroxypropanoic acid	1.0142	Procyanidin B1	0.0276
12(13)-DiHOME	0.9835	2-(Acetylamino)hexanoic acid	0.0253
1,2,3-cyclopropanetricarboxylic acid	0.8778	Apigetrin	0.0233
9-HpODE	0.8557	4-(beta-D-Glucopyranosyloxy)-2-methylenebutanoic acid	0.0193
4-Oxoproline	0.8286	4,4'-Sulfonylbis[2-(prop-2-en-1-yl)phenol]	0.0183
3-Anisic acid	0.7156	(2E)-4-Hydroxy-3,7-dimethyl-2,6-octadien-1-yl beta-D-glucopyranoside	0.0166
N-{4-[(R)-[(3R)-4-(4-Fluorobenzyl)-5-oxo-3-morpholinyl](hydroxy)methyl]phenyl}acetamide	0.7088	8(S),15(S)-DiHETE	0.0165
Catechin	0.6399	12-Hydroxy-10-(hydroxymethyl)-6-methyl-2-(4-methyl-3-penten-1-yl)-2,6,10-dodecatrienoic acid	0.0145
trans-Aconitic acid	0.6325	3-[(1-Carboxyvinyl)oxy]benzoic acid	0.0140
Methylsuccinic acid	0.6277	N-[(1S,3aS,5S,7aR)-5-Hydroxy-7a-{3-[4-(2-methoxyphenyl)-1-piperazinyl]-3-oxopropyl}-3,3,5-trimethyloctahydro-1H-inden-1-yl]nicotinamide	0.0136
Oryzalin	0.5017	NP-019498	0.0131
6-Hydroxycaproic acid	0.4620	NP-012551	0.0115
Glutaric acid	0.4571	NP-020174	0.0102
NP-008309	0.4380	4-Butyl-N-(3-cyano-2-thienyl)benzamide	0.0100
2-Methylbutyl beta-D-glucopyranoside	0.4203	[(3S,4R,5S)-5-{[(2S,3R,4S,5S,6R)-4,5-dihydroxy-6-(hydroxymethyl)-2-(4-hydroxyphenoxy)oxan-3-yl]oxy}-3,4-dihydroxyoxolan-3-yl]methyl 4-hydroxybenzoate	0.0099
Gallic acid	0.3962	3,5-Dihydroxy-2-(4-hydroxyphenyl)-4-oxo-3,4-dihydro-2H-chromen-7-yl hexopyranoside	0.0088
D-(+)-Malic acid	0.3396	12-Epileukotriene B4	0.0088
9,10-dihydroxy-12Z-octadecenoic acid	0.3013	Arjungenin	0.0083
L-Iditol	0.2943	(2E)-4-Hydroxy-4-{4-hydroxy-2-[(1E)-6-hydroxy-1-hepten-1-yl]cyclopentyl}-2-butenoic acid	0.0068
2,5-Dihydroxybenzaldehyde	0.2713	7-Hydroxy-2-(4-hydroxyphenyl)-4-oxo-3,4-dihydro-2H-chromen-5-yl β-D-glucopyranoside	0.0068
NP-020139	0.2538	3-Hydroxybenzyl alcohol	0.0065
2-Furoic acid	0.2184	(2E)-5-hydroxy-N-[3-(5-{3-[(2E)-5-hydroxy-3-methylpent-2-enamido]propyl}-3,6-dioxopiperazin-2-yl)propyl]-3-methylpent-2-enamide	0.0062
Neochlorogenic acid	0.2180	3-Methylxanthine	0.0060
2,4,6-Trihydroxyacetophenone	0.1954	Astragalin	0.0058
2-Hydroxy-2-methyl-3-buten-1-yl beta-D-glucopyranoside	0.1809	(3β,5ξ,9ξ)-3,23-Dihydroxy-1-oxoolean-12-en-28-oic acid	0.0054
NP-018716	0.1668	NP-003191	0.0048
3-tert-Butyladipic acid	0.1629	5-Chlorosalicylic acid	0.0048

NP-020205	0.1610	4-[4-(4-Hydroxy-3-methoxyphenyl)tetrahydro-1H,3H-furo[3,4-c]furan-1-yl]-2-methoxyphenyl hexopyranoside	0.0045
1-(Carboxymethyl)cyclohexanecarboxylic acid	0.1340	1-Phenyl-1H-pyrazolo[3,4-d]pyrimidin-4-amine	0.0038
1-(4-fluorophenyl)-2-(4-methoxyphenyl)-4-(2-naphthyl)butane-1,4-dione	0.1325	13-HODE	0.0033
NP-020916	0.1169	N-Acetyl-L-tyrosine	0.0031
NP-013285	0.1169	4,5-Dicaffeoylquinic acid	0.0027
4-(hydroxymethyl)benzoic acid	0.1158	3-Hydroxybutyric acid	0.0027
Pimelic acid	0.1134	Chlorogenic acid	0.0025
Kaempferol-3-Glucuronide	0.1094	α-Cyano-3-hydroxycinnamic acid	0.0024
Pyrogallol	0.1088	(1S,3R,4R,5R)-1,3,4-trihydroxy-5-{[(2E)-3-(4-hydroxy-3-methoxyphenyl)prop-2-enoyl]oxy}cyclohexane-1-carboxylic acid	0.0019
Lariciresinol 4-O-glucoside	0.0996	1,3,7-Trimethyluric acid	0.0017
D-(-)-Quinic acid	0.0966	2-Hydroxymyristic acid	0.0016
N-Acetyl-DL-tryptophan	0.0921	(3R,5R)-1,3,5-Trihydroxy-4-{[(2E)-3-(4-hydroxy-3-methoxyphenyl)-2-propenoyl]oxy}cyclohexanecarboxylic acid	0.0014
2,3-dinor-8-iso Prostaglandin F2?	0.0763	4-Aminosalicylic acid	0.0013
Terephthalic acid	0.0680	3-Fluoro-N-({(1S,4S,6S)-6-isopropyl-3-methyl-4-[2-oxo-2-(2-propyn-1-ylamino)ethyl]-2-cyclohexen-1-yl}methyl)benzamide	0.0012
3-Hydroxyphenylacetic acid	0.0656	2-(4-Hydroxyphenyl)ethyl 6-O-[(2R,3R,4R)-3,4-dihydroxy-4-(hydroxymethyl)tetrahydro-2-furanyl]-beta-D-glucopyranoside	0.0012
NP-018661	0.0596	5-[({(2R,4S,5R)-5-[1-Methyl-3-(2-thienyl)-1H-pyrazol-5-yl]-1-azabicyclo[2.2.2]oct-2-yl}methyl)amino]-5-oxopentanoic acid	0.0008
4-Hydroxybenzaldehyde	0.0588	13,14-Dihydro-15-keto Prostaglandin A2	0.0008
Quercetin-3β-D-glucoside	0.0527	Resolvin E1	0.0008
Catechol	0.0519	Rutin	0.0008
Cynaroside	0.0492	2,4,6-Trihydroxy-2-(4-hydroxybenzyl)-1-benzofuran-3(2H)-one	0.0007
13,14-Dihydro-15-keto-tetranor prostaglandin F1α	0.0464	Linoleic acid-biotin	0.0007
NP-005196	0.0450	4-Methylphenol	0.0007
Tetradecanedioic acid	0.0440	Oleic acid-biotin	0.0006
12(13)-DiHOME	0.0433	NP-003535	0.0004

Figure S1. Metabolic activity of Neo-dermal fibroblast cells after 48 h of seeding in presence of different concentration (from 1000 to 100 µg/mL) of the metabolites mix. The results are shown as average ± SD after normalisation to the control of cells seeded on TCPs (**A**); Live/Dead images of Neo-dermal fibroblast cells after 48 h of seeding in presence of different concentration metabolites mix: 100 (**B**), 250 (**C**), 500 (**D**) and (**E**) 1000 µg/mL. Scale bar = 300 µm.

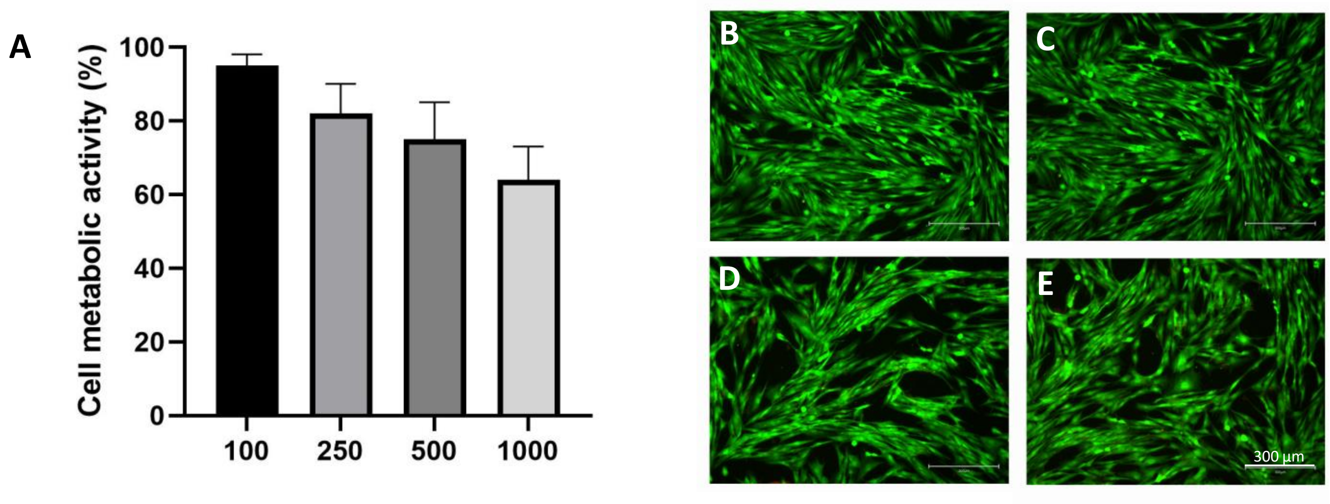

Figure S2. Fluorescent images of the repressive effect of ROS activity after H_2O_2-induced intracellular oxidative stress on Neo-dermal fibroblasts (**A**) and different concentration metabolites mix: 10, 50, 100 and 500 µg/mL (**C**). Fluorescent image of the fibroblasts without H_2O_2-induced intracellular oxidative stress are used as control (**B**). Scale bar = 300 µm.

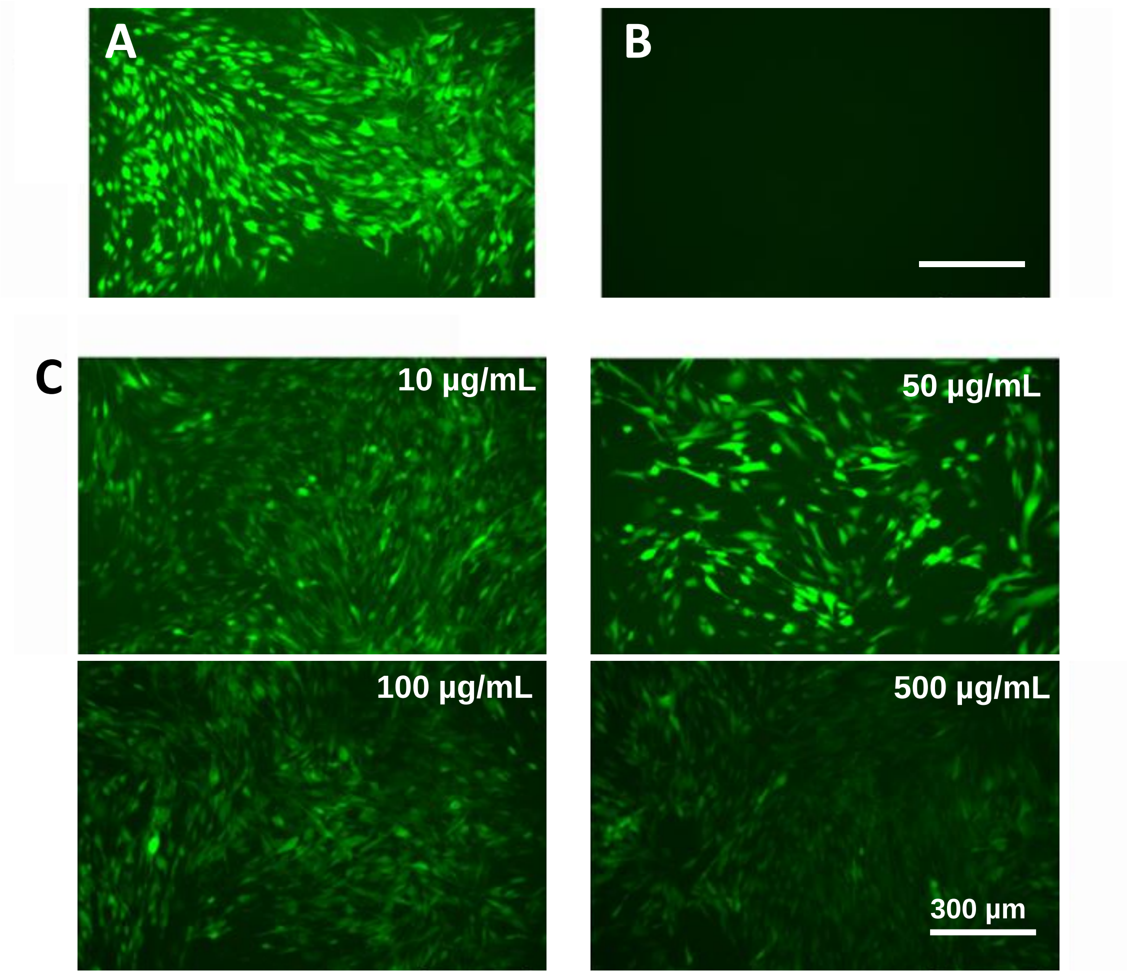

Supplement: NA-006-D4NA00248B-s001 [file NA-006-D4NA00248B-s001.pdf]
